# The Association of eHealth Literacy Skills and mHealth Application Use Among US Adults With Obesity: Analysis of Health Information National Trends Survey Data

**DOI:** 10.2196/46656

**Published:** 2024-01-10

**Authors:** George Shaw Jr, Bianca A Castro, Laura H Gunn, Keith Norris, Roland J Thorpe Jr

**Affiliations:** 1 Department of Public Health Sciences School of Data Science University of North Carolina at Charlotte Charlotte, NC United States; 2 Department of Public Health Sciences University of North Carolina at Charlotte Charlotte, NC United States; 3 The University of California Los Angeles Division of General Internal Medicine and Health Services Research University of California, Los Angeles Los Angeles, CA United States; 4 Department of Health, Behavior and Society Johns Hopkins University Baltimore, MD United States

**Keywords:** accessibility, eHealth literacy, mHealth, multivariable logistic regression, obesity, smartphones

## Abstract

**Background:**

Physical inactivity and a poor diet are modifiable behaviors that contribute to obesity. Obesity is a well-recognized risk factor for chronic diseases, including diabetes. Mobile health (mHealth) apps can play an important adjuvant role in preventing and treating chronic diseases and promoting positive health behavior change among people with obesity, and eHealth literacy skills have the potential to impact mHealth app use.

**Objective:**

The purpose of this study was to explore the associations between the 2 dimensions, access and application, of eHealth literacy skills and mHealth app use among US adults (≥18 years of age) with obesity (BMI ≥30 kg/m^2^).

**Methods:**

Data were obtained from February to June 2020 using the Health Information National Trends Survey 5. A total of 1079 respondents met the inclusion criteria of adults with obesity and owners of smartphones. Individual associations between mHealth app use and sociodemographic variables were explored using weighted chi-square and 2-tailed *t* tests. A multivariable weighted logistic regression model was fitted, and adjusted odds ratios (ORs) of using mHealth apps with corresponding 95% CIs were reported across multiple sociodemographic variables. An Ising model-weighted network visualization was produced. A receiver operating characteristic curve was calculated, and the area under the curve was reported with the corresponding Delong 95% CI.

**Results:**

A majority of respondents were female (550/923, 59.6%) or non-Hispanic White (543/923, 58.8%). Individuals in households earning less than US $50,000 comprised 41.4% (382/923) of the sample. All sociodemographic variables were found to be univariately significant at the 5% level, except employment and region. Results from the multivariable weighted logistic regression model showed that the adjusted odds of using an mHealth app are 3.13 (95% CI 1.69-5.80) and 2.99 (95% CI 1.67-5.37) times higher among those with an access eHealth literacy skill of using an electronic device to look for health or medical information for themselves and an application eHealth literacy skill of using electronic communications with a doctor or doctor’s office, respectively. Several sociodemographic variables were found to be significant, such as education, where adjusted ORs comparing subgroups to the lowest educational attainment were substantial (ORs ≥7.77). The network visualization demonstrated that all eHealth literacy skills and the mHealth app use variable were positively associated to varying degrees.

**Conclusions:**

This work provides an initial understanding of mHealth app use and eHealth literacy skills among people with obesity, identifying people with obesity subpopulations who are at risk of a digital health divide. Future studies should identify equitable solutions for people with obesity (as well as other groups) and their use of mHealth apps.

## Introduction

### Overview

Physical inactivity and poor dietary behaviors are modifiable behaviors that contribute to obesity [1,2]. Recent studies show that obesity affects nearly 42% of the US population aged 20 years or older [3], with an associated excess annual estimated medical cost of upwards of US $170 billion in 2019 [4]. Obesity is a well-recognized risk factor for chronic diseases, including diabetes, cardiovascular diseases, and cancer, and a significant cause of premature morbidity and mortality [2,5]. Wang et al [6] demonstrated the importance of reducing the weight of patients classified as obese, which is a major contributor to the increased incidence of type 2 diabetes mellitus. Obesity is a complex and multifaceted disease, extending far beyond the realm of individual behavior or mere lifestyle choices. Genetic, environmental, and socioeconomic factors in the development of obesity [7]. However, mobile health (mHealth) apps can play an important adjuvant role in preventing and treating chronic diseases and promoting positive health behavior change among individuals with obesity [8,9], with several factors influencing users’ acceptance of and engagement with mHealth apps [10-12]. Previous intervention studies have used mHealth apps to promote health behavior change among individuals with obesity [13,14]. Participants from the reference studies found that the use of mHealth made the intervention helpful and benefited weight loss when used in conjunction with other weight loss intervention methods. mHealth apps provide access to health information and can extend this access to underserved groups, particularly those at higher risk of chronic diseases [15]. However, many individuals with chronic diseases like obesity fail to engage in mHealth app use [16].

### mHealth and eHealth Literacy

Istepanian et al [17] defined mHealth as mobile computing, medical sensors, or communication technologies designed for health care. Recent interventions involving mHealth apps provide evidence of improvement in participants’ self-care and disease self-management [18,19]. It is estimated that more than 350,000 mHealth apps are available on the market [20]. Despite the number of digital health interventions targeting weight management, the level to which users actively and regularly engage with those apps entails user engagement early in the intervention design process [21]. While health literacy is a broader concept associated with the ability of individuals to obtain and understand health information to make rational health decisions, eHealth literacy comprises the complex navigation of health care information from internet sources [22,23]. Kontos et al [24] showed that people with lower levels of education were less likely to use the internet to communicate with a doctor or use health information on their mobile devices. Moreover, national and international studies have shown that weight-management apps are beneficial for improving weight loss [25,26]. Previous studies have recommended focusing on understanding the level of health literacy of recipients who may use these apps, particularly those interested in weight loss interventions. Understanding eHealth literacy skills is critical when evaluating health information from mHealth apps and the subsequent application of the knowledge gained [27,28].

eHealth literacy is defined as seeking, finding, understanding, and appraising health information from electronic sources and applying the knowledge acquired to address or solve a health-related problem [29,30]. More recently, eHealth literacy has been conceptualized in dimensions including access to digital services and the application of services and information that satisfy users [31]. People lacking or with a low level of health literacy or eHealth literacy skills benefit less from digital health information and health informatics interventions [32,33], as low eHealth literacy skills were found to impact mHealth app use when mediated through mHealth app efficacy [23]. By contrast, those with higher levels of health literacy and eHealth literacy skills report a positive connection between mHealth app use and health outcomes [34].

mHealth apps are operated across a diverse group of users. This includes individuals with varying incomes, ages, races, ethnicities, and educations [35]. Curating data that include this and other defining personal characteristics require a significant number of resources. Few studies of eHealth literacy and mHealth app use have included a diverse group of participants. Through a national data set of noninstitutionalized adults, this study uses a diverse national data set. The purpose of this study is to explore the potential associations between the 2 dimensions, access and application, of eHealth literacy skills and mHealth app use among a diverse group of US adults (aged ≥18 years) with obesity (BMI ≥30 kg/m^2^).

### Theoretical Underpinning

Seeking health information has become a typical behavior among people of all ages and health conditions [36]. The information obtained when factoring in a person’s eHealth literacy skills, has the potential to influence health outcomes. This is particularly important for people with obesity. People with obesity have lower self-confidence in managing their health. However, people with obesity view communication with their physician as helpful with self-care weight management [37]. Therefore, people with obesity may improve their confidence over time in managing their weight if they have the eHealth literacy skills to seek information from their physician electronically. There have been numerous information behavior theories and models developed to understand how individuals seek and use information [38]. For example, Zare-Farashbandi and Lalazaryan [39] designed their health information acquisition model based on 6 stages of information seeking. The model acknowledges that the information-seeking process can be iterative and that there is a need for a feedback loop in the search process. However, the model does not consider personal or contextual factors affecting information seeking. Longo’s model of health information considers the effects of personal and contextual factors on the information-seeking behavior of patients [40]. Focusing on patients with chronic diseases, the model was significant in depicting the output process of information-seeking for patients [39]. These theories and models have also considered various social contexts and population groups, such as older individuals, patients with cancer, prisoners, and diabetics [38,41-43]. However, this study is informed by Lenz’s [41] Information Seeking Model, which is the foundation of many fundamental tenets of recent models and instruments aimed at measuring eHealth literacy skills.

According to this framework, in which information gathering is part of the decision-making process, individuals follow 6 stages to seek health information. First, they receive a stimulus from their previous disease experience or the environment. Second, they establish their informational goals, including sources, time available, and the type of information needed. Third, the person decides whether or not to actively access the information they want. The decision is based on the individual’s previous knowledge, background, and the expected cost-benefit of the action. The fourth stage is of particular interest, characterized by the information-seeking action itself. This step is correlated to the eHealth literacy dimension of access, and it could be an in-depth search or superficial information gathering, depending on the person’s need and previous attempts. The access dimension corresponds to having the availability of digital services that suit people’s needs and work correctly [44]. The fifth stage corresponds to information achievement and interpretation. This step is related to the eHealth literacy dimension of application, in which the individual understands and appraises or applies the information obtained. In this final stage, people may have to decide on the adequacy of the acquired information [39]. Understanding health information seeking through the potential associations of eHealth literacy skills and mHealth app use may provide insights into how population groups with health disparities with chronic conditions such as obesity can access and apply the information they seek [45]. Using this framework, the objective of this study is to explore the potential associations between the 2 dimensions, access and application, of eHealth literacy skills and mHealth app use among a diverse group of US adults with obesity.

## Methods

### Data

The Health Information National Trends Survey (HINTS) was used to explore the potential association between mHealth app use and eHealth literacy skills. HINTS has been administered every few years by the National Cancer Institute since 2003, and the data sets that have been made publicly available are used for evaluating health information access and use among US adults [24,46,47]. HINTS collects representative data about noninstitutionalized US adults’ knowledge, access, attitudes, and use of cancer- and health-related information. The survey uses a 2-stage stratified random sampling that selects households from residential addresses in the United States and then selects 1 adult within each household [24,46].

This study used the HINTS 5, Cycle 4 data set. The data were collected from February to June 2020 and comprised responses from 3865 participants. Despite the COVID-19 impact on society, the response rate for the survey remained high. The response rate (37%) for the survey remained relatively high and was even higher than prepandemic HINTS 5 surveys, which experienced response rates of at most 33% [48]. However, COVID-19 impacted the time frame in which the data are typically collected. Individuals included in this analysis were those who indicated ownership of a tablet, smartphone, or both and self-declared a BMI ≥30 kg/m^2^ (obese). The dependent variable was based on respondents’ answers to the following item: “In the past 12 months, have you used any of these health or wellness apps?” The binary variable derived was used to indicate those who reported using any health or wellness apps within the past 12 months and those who did not.

The main independent variables representing eHealth literacy skill’s access and application dimensions were the following four items pertaining to eHealth information and services, connecting to the common stem of “In the past 12 months, have you used a computer, smartphone, or other electronic means to do any of the following: (1) looked for health or medical information for yourself; (2) used email or the internet to communicate with a doctor or doctor’s office; (3) looked medical test results; and (4) made appointments with a health care provider?” Access within this context is the information-searching behavior involved in accessing information. Application within this context is defined as the interpretation and appraisal of information aimed at completing an action. Additional covariates extracted include age in years, health insurance status, sex at birth, employment status, marital status, education, annual household income (in ranges), race and ethnicity, and US Census region. These variables have been used in previous studies to evaluate mHealth app use or can be relevant confounders regarding the associations between eHealth literacy skills and mHealth app use [49,50].

Due to low counts, the following categories were combined: employment status of unemployed across lengths of unemployment; employment status of students and others; marital status of separated and divorced; marital status of married and those living as married or with a romantic partner; education categories below 11 years of education; and race and ethnicity categories of non-Hispanic Native Hawaiian or Other Pacific Islander and American Indian or Alaska Native.

### Statistical Analysis

In order to investigate the research objective and hypothesis, a comprehensive statistical analysis was performed on the collected data using univariate and multiple logistic regression modeling. The weights provided by HINTS were used to perform all analyses and adjust for sampling biases [51]. A weighted complete case analysis was performed on the data. Characteristics were summarized using means, SDs, counts, and percentages as appropriate. Weighted chi-square and 2-tailed *t* tests were used to explore univariate associations between each of the covariates and mHealth app use, with test statistics and corresponding *P* values tabulated. Visualizations were created to explore associations, including (1) Ising model network weighted analysis of the associations between the main independent variables (eHealth literacy skill’s access and application covariates) and the outcome; (2) weighted box plot for the continuous covariate (age) and the dependent variable; and (3) multiple weighted 100% stacked bar charts across the main independent variables and the dependent variable. Additional weighted 100% stacked bar charts were constructed (Multimedia Appendix 1) to visualize the sociodemographic variables and the outcome.

The primary study aim is to assess associations between mHealth app use (binary outcome) and each of the eHealth literacy skills dimensions of access and application (main covariates). Univariate analysis is included to provide a comprehensive description of the individual variables in the study and establish a foundation for more complex multivariable analyses. These were further examined using a multivariable weighted logistic regression adjusted for the aforementioned sociodemographic factors. Adjusted odds ratios (ORs), corresponding 95% CIs, and *P* values were reported across eHealth literacy skills dimensions and sociodemographic variables. Results were tabulated and highlighted using a significance level of 5%. A pseudo-*R*^2^ was calculated. The receiver operating characteristic (ROC) is a common approach used to measure the sensitivity versus specificity of logistic models. Additionally, the area under the curve (AUC) is a single metric for that trade-off, with AUC=1 meaning that the model perfectly fits the data and AUC=0.5 indicating there is a split chance that the model fits the data. Both of these approaches are used to evaluate the performance of logistic models. The ROC curve was estimated, and the corresponding AUC value and Delong 95% CI were reported. R software (version 4.0.3; R Foundation for Statistical Computing) was used for statistical analyses.

### Ethical Considerations

This research was approved by the institutional review board (IRB) of the University of North Carolina at Charlotte (study #IRB-22-0585). This data set consisted of deidentified, aggregated data. The IRB approval process did not require additional consent from the respondents representing the data.

## Results

A total of 1079 participants were identified as obese and owners of a smartphone, tablet, or both. Fewer than 15% (156/1079) of the responses were removed due to incomplete or incoherent data, resulting in 923 complete observations, with mHealth app use (dependent variable), eHealth literacy skills dimensions (main independent variable), and additional covariates summarized in Table 1. The average age was 53.51 (SD 14.91) years, and most participants were female (550/923, 59.6%) and non-Hispanic White (543/923, 58.8%). A college degree or above was the highest level of education for 43.3% (400/923) of study participants, and they were mainly employed (with a single or multiple employer; 594/923, 64.4%) and covered by health insurance (872/923, 94.5%). The South contained the largest percentage of participants (436/923, 46.7%), which also corresponds to the nation’s most populous region [52]. Individuals in households earning less than US $50,000 comprised 41.4% (382/923) of the sample, and 18.9% (174/923) of participants had an annual household income that fell within the range, containing the median annual household income in the United States of US $67,521 in 2020 [53].

The majority of participants (482/923, 52.2%) did not use mHealth apps, resulting in a balanced outcome variable. Within the eHealth literacy skills access dimension, 77.5% (715/923) of respondents used an electronic device to look for health or medical information for themselves within the past 12 months, and approximately half (468/923, 50.7%) used electronic means to look up medical test results, also within the past 12 months. Within the eHealth literacy skills application dimension, 55.6% (513/923) of respondents used email or the internet to communicate with a doctor or doctor’s office within the past 12 months, and 53.3% (429/923) made an appointment with a health care provider through electronic means in that same time period. We also examined the univariate association between mHealth app use, covariates, and main independent covariates.

**Figure 1:**
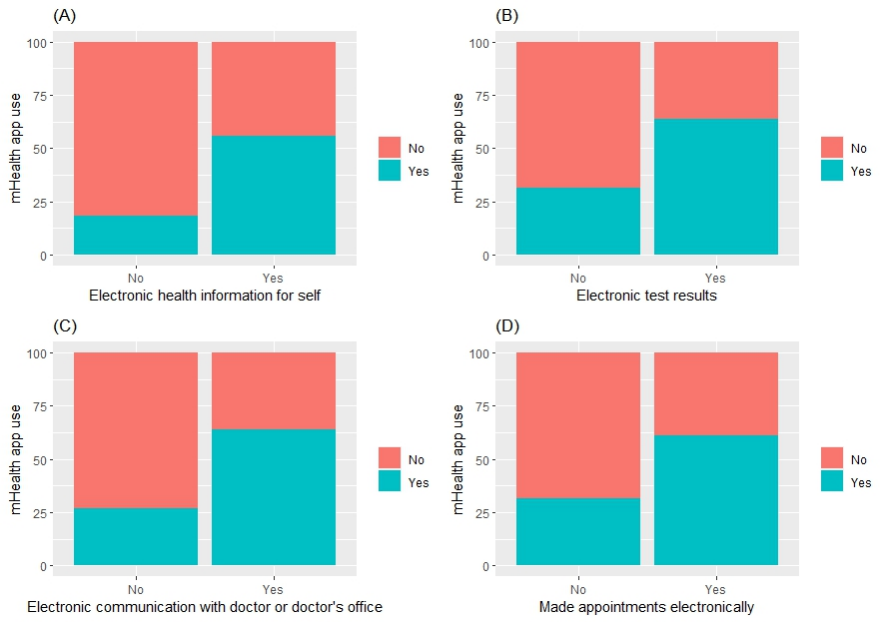
Visualization of weighted 100% stacked bar charts for each of the eHealth literacy skills dimensions (main covariates) against mHealth app use (outcome). A: Electronic health information for self; B: Electronic test results; C: Electronic communication with doctor or doctor's office; D: Made appointments electronically.

**Figure 2:**
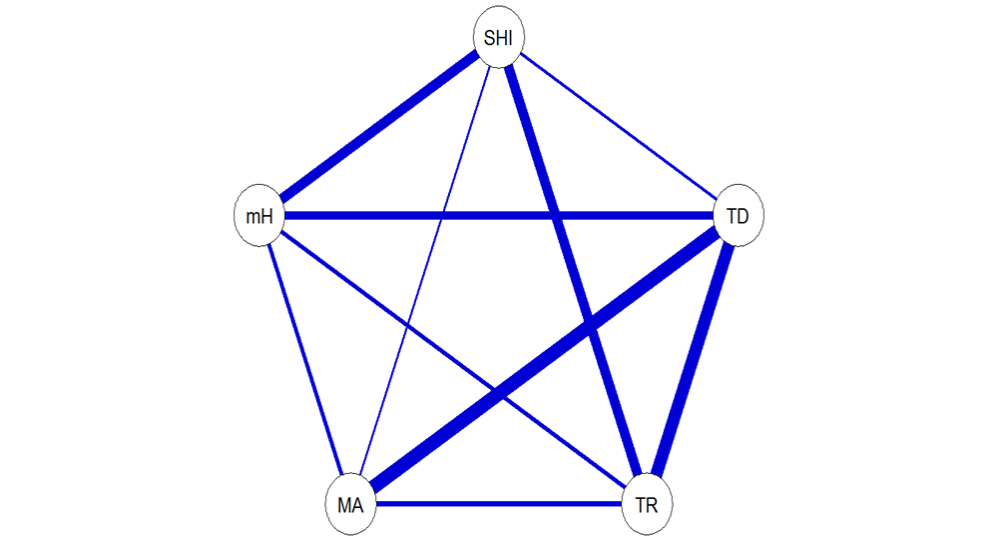
Ising model-weighted network visualization of eLASSO associations (unadjusted by other covariates and with 0.25 penalization factor) between the eHealth literacy skills dimensions (SHI: self-health information; TD: talk to a doctor or doctor’s office; TR: test results; MA: made appointments; and mH: mHealth app use). Thicker edges (lines) between nodes (circles) represent stronger associations.

Table 2 summarizes results from weighted chi-square and *t* tests for univariate associations between mHealth app use and each of the covariates. Most covariates and all the main covariates were found to be significant at the 5% level. All eHealth literacy skills dimensions were found to be significantly associated with mHealth app use based on univariate weighted chi-square tests (*P*<.001). Similarly, all demographic factors were found to be significantly associated with mHealth app use except for employment status (*P*=.20) and Census region (*P*=.16). Figure 1 displays pairwise weighted 100% stacked bar charts for each of the eHealth literacy skills dimensions versus mHealth app use. Figure 2 portrays a joint network representation of the weighted associations between the eHealth literacy skills dimensions and mHealth app use, which demonstrate strong positive associations both between the skills dimensions as well as between those and the outcome (mHealth app use). Figures S1-S9 in Multimedia Appendix 1 include a weighted box plot (age) and weighted 100% stacked bar charts visualizing the univariate associations with mHealth app use.

Table 3 presents the results of the multivariable weighted logistic regression model. The adjusted odds of using an mHealth app are 3.13 (95% CI 1.69-5.80) times higher among those who responded with an access eHealth literacy skill of using an electronic device to look for health or medical information for themselves within the past 12 months. Similarly, those with an application eHealth literacy skill of using email or the internet to communicate with a doctor or doctor’s office within the past 12 months experience 2.99 (95% CI 1.67-5.37) times higher odds of using an mHealth app compared to those without this skill. Sociodemographic factors found to be significantly associated with mHealth app use include age, disabled or retired status, single or never married or widowed, education, and Hispanic ethnicity. Each additional year of age is associated with 4% lower odds of using mHealth apps (OR 0.96, 95% CI 0.94-0.98). Disabled and retired participants experienced 4.21 (95% CI 1.28-13.82) and 2.53 (95% CI 1.14-5.60) higher odds, respectively, of using mHealth apps compared to those who were employed. Single or never married and widowed participants experienced 49% and 81% lower odds of mHealth app use, respectively, than those who are married, living as married, or living with romantic partners. Previous work has indicated that surrogate seekers, those who may seek health information on behalf of others, were more likely to be married or have someone close to them with a chronic illness [54]. Those who received more than 11 years of formal education experienced higher odds of mHealth app use than those with 11 years or less, with OR estimates ranging from 7.77 to 17.24, though with substantially wide CIs. Hispanic participants experienced higher odds of using mHealth apps than non-Hispanic White participants (OR 2.61, 95% CI 1.28-5.33). Insurance status, sex at birth, annual household income, and Census region were not found statistically significant upon adjusting for the other covariates, though there is some level of collinearity present among sociodemographic covariates, as demonstrated in the univariate significance of some of these variables. The multivariable weighted logistic regression adjusted for sociodemographic characteristics showed relatively strong explanatory power with a pseudo-*R*^2^ of 0.32 and AUC of 0.7957 (95% CI 0.7671-0.8243). The corresponding ROC is included in Multimedia Appendix 1.

**Table 1:** Unweighted characteristics of study participants (n=923) using the 2020 Health Information National Trends Survey data set.

Sociodemographic variables						Values
Age (years), mean (SD)						53.51 (14.91)
**Health insurance, n (%)**						
	Insured					872 (94.5)
	Uninsured					51 (5.5)
**Sex at birth, n (%)**						
	Female					550 (59.6)
	Male					373 (40.4)
**Employment status, n (%)**						
	Disabled					67 (7.3)
	Employed					494 (53.5)
	Homemaker					25 (2.7)
	Multiple					100 (10.8)
	Retired					183 (19.8)
	Unemployed					42 (4.6)
	Other					12 (1.3)
**Marital status, n (%)**						
	Married or living as married or with a romantic partner					518 (56.1)
	Separated or divorced					180 (19.5)
	Single or never married					159 (17.2)
	Widowed					66 (7.2)
**Education, n (%)**						
	≤11 years					49 (5.3)
	12 years or completed high school					174 (18.9)
	Post–high school training other than college (vocational or technical)					68 (7.4)
	Some college					232 (25.1)
	College graduate					241 (26.1)
	Postgraduate					159 (17.2)
**Annual household income (US $), n (%)**						
	0-9999					51 (5.5)
	10,000-14,999					49 (5.3)
	15,000-19,999					37 (4)
	20,000-34,999					113 (12.2)
	35,000-49,999					132 (14.3)
	50,000-74,999					174 (18.9)
	75,000-99,999					128 (13.9)
	100,000-199,999					198 (21.5)
	≥200,000					41 (4.4)
**Race and ethnicity, n (%)**						
	Black or African American					148 (16)
	Hispanic					171 (18.5)
	Non-Hispanic Asian					18 (2)
	Non-Hispanic Native Hawaiian or Other Pacific Islander or American Indian or Alaska Native					9 (1)
	Non-Hispanic White					543 (58.8)
	Non-Hispanic multiple races					34 (3.7)
**Census region, n (%)**						
	Midwest					155 (16.8)
	Northeast					141 (15.3)
	South					431 (46.7)
	West					196 (21.2)
**Outcome variable, n (%)**						
	**mHealth app use**					
		No			482 (52.2)	
		Yes			441 (47.8)	
**Main covariates, n (%)**						
	**eHealth literacy skills access dimension**					
		**Electronic health information for self**				
			No	208 (22.5)		
			Yes	715 (77.5)		
		**Electronic test results**				
			No	455 (49.3)		
			Yes	468 (50.7)		
	**eHealth literacy skills application dimension**					
		**Electronic communication with doctor or doctor’s office**				
			No	410 (44.4)		
			Yes	513 (55.6)		
		**Made provider appointments electronically**				
			No	431 (46.7)		
			Yes	492 (53.3)		

**Table 2:** Weighted chi-square and 2-tailed *t* tests (test statistics and *P* values) for univariate associations between mHealth app use (dependent variable) and each of the covariates.

Variable				Chi-square (*df*)		*P* value
**Sociodemographic factors**						
	Age (years)			3.77 (921)^a^		<.001
	Health insurance			6.28 (1)		.01
	Sex at birth			6.25 (1)		.01
	Employment status			8.54 (6)		.20
	Marital status			18.55 (3)		<.001
	Education			78.02 (5)		<.001
	Annual household income			34.38 (8)		<.001
	Race and ethnicity			11.31 (5)		.046
	Census region			5.18 (3)		.16
**Main covariates**						
	**eHealth literacy skills: access dimension**					
		eHealth information for self	95.60 (1)		<.001	
		Electronic test results	97.48 (1)		<.001	
	**eHealth literacy skills: application dimension**					
		Electronic communication with doctor or doctor’s office	127.87 (1)		<.001	
		Made provider appointments electronically	81.48 (1)		<.001	

^a^*t* test was used for the univariate analysis.

**Table 3:** Adjusted odds ratios (ORs), corresponding 95% CIs, and *P* values for the multivariable weighted logistic regression model assessing mHealth app use (n=923). The regression model included adjustments for eHealth literacy, age, insurance status, sex, employment and marital status, education, income, race and ethnicity, and census region.

Characteristics			OR (95% CI)	*P* value
**Explanatory demographic variables**				
	Age (years)		0.96 (0.94-0.98)	<.001
	Insured		2.25 (0.94-5.38)	.07
	Male		0.75 (0.46-1.20)	.22
	**Employment status**			
		Employed (reference)	N/A^a^	N/A
		Disabled	4.21 (1.28-13.82)	.02
		Homemaker	2.10 (0.66-6.70)	.21
		Multiple	2.16 (0.92-5.09)	.08
		Retired	2.53 (1.14-5.60)	.02
		Unemployed	1.12 (0.44-2.87)	.81
		Other	0.21 (0.04-1.12)	.07
	**Marital status**			
		Married or living as married or with a romantic partner (reference)	N/A	N/A
		Separated or divorced	0.67 (0.34-1.33)	.25
		Single or never married	0.51 (0.27-0.96)	.04
		Widowed	0.19 (0.06-0.57)	.003
	**Education**			
		≤11 years (reference)	N/A	N/A
		12 years or completed high school	7.77 (2.08-29.01)	.002
		Post–high school training other than college	12.75 (3.18-51.17)	<.001
		Some college	9.25 (2.60-32.98)	<.001
		College graduate	14.01 (3.68-53.26)	<.001
		Postgraduate	17.24 (4.09-72.64)	<.001
	**Annual household income (US $)**			
		<10,000 (reference)	N/A	N/A
		10,000-14,999	1.67 (0.38-7.45)	.50
		15,000-19,999	0.81 (0.20-3.18)	.76
		20,000-34,999	1.31 (0.41-4.26)	.65
		35,000-49,999	2.47 (0.73-8.37)	.15
		50,000-74,999	2.27 (0.71-7.27)	.17
		75,000-99,999	3.16 (0.90-11.04)	.07
		100,000-199,999	2.47 (0.72-8.40)	.15
		≥200,000	1.81 (0.37-8.93)	.47
	**Race and ethnicity**			
		Black or African American	1.05 (0.51-2.15)	.90
		Hispanic	2.61 (1.28-5.33)	.008
		Non-Hispanic Asian	0.30 (0.06-1.50)	.14
		Non-Hispanic Native Hawaiian or Other Pacific Islander or American Indian or Alaska Native	1.64 (0.17-16.19)	.67
		Non-Hispanic White (reference)	N/A	N/A
		Non-Hispanic multiple races	1.21 (0.34-4.22)	.77
	**Census region**			
		South (reference)	N/A	N/A
		Midwest	1.42 (0.73-2.75)	.30
		Northeast	0.89 (0.47-1.69)	.72
		West	1.09 (0.57-2.08)	.80
	Intercept		0.03 (0.00-0.19)	<.001
	**eHealth literacy skills: access dimension**			
		Electronic health information for self (reference: yes)	3.13 (1.69-5.80)	<.001
		Electronic test results (reference: yes)	1.55 (0.87-2.73)	.13
	**eHealth literacy skills: application dimension**			
		Electronic communication with a doctor or doctor’s office (reference: yes)	2.99 (1.67-5.37)	<.001
		Made appointments electronically (reference: yes)	1.53 (0.91-2.58)	.11

^a^N/A: not applicable.

## Discussion

### Overview

The purpose of this study was to explore the associations between the 2 dimensions, access and application, of eHealth literacy skills and mHealth app use among US adults with obesity. We used the HINTS 2020 data to explore this potential association with a sample of 923 respondents with complete information represented in the data set. We found that the majority of the respondents had health insurance, were female, and were non-Hispanic White, with an average age of 54 years. Also, more than half of the respondents had some level of college or were college graduates. This study highlights the association between eHealth literacy skills for accessing and the application of health information using mHealth apps among people with obesity.

The weighted univariate analyses demonstrated associations between all of the covariates and mHealth app use except employment status and census region. Socioeconomic factors of education and income have been found to be important in the general use of content within digital environments (ie, internet) [44]. However, more specifically to this study, these factors are important in showing the potential relationship they have with mHealth app use among people with obesity. When considering weight management or physical activity interventions using mHealth apps, future interventions should attempt to improve the eHealth literacy of participants by targeting segments of people with obesity identified to be more at risk, such as older individuals with obesity or those in lower income brackets. These initial metrics can be collected through a variety of eHealth literacy assessment tools. The eHealth Literacy Scale, for example, has been studied in diverse languages and populations, and it was designed to convey an estimate of people’s eHealth-related skills. Other instruments, such as the eHealth Literacy Questionnaire, were established to support researchers, designers, and the government in evaluating, developing, and applying effective digital health interventions [54]. Previous research identified that patients with adequate eHealth literacy had more ability to seek health information on the internet and find reliable and high-quality information than patients with inadequate eHealth literacy [55,56].

Accessing health information requires active information-seeking skills. Additionally, context and behaviors to gain information are intertwined in this process. Respondents’ access to web-based health information (seeking health information for themselves) can be informed by the Lenz search behavior stage. The respondents’ access to information requires a search for information from impersonally related sources. There is no indication of the familiarity that respondents have with these resources based on the survey questions. People with obesity who use mHealth devices may exhibit multiple factors in their search for health information, and recent eHealth literacy work supports the nuances involved in seeking health information [57,58].

The information acquired through the information-seeking process impacts an information seeker’s decision-making process. Electronic communication with the doctor’s office can be the result of gathering enough information to move forward based on their original goal or a stop in the information acquisition process that prompts information seeking through a personal connection through digital communication. The results from this work are not intended to model these variables but demonstrate that Lenz’s model, enhanced with recent theories, may help inform studies aimed at understanding active information seeking at the intersection of digital health devices such as mHealth and eHealth literacy skills. Recent models and theories commonly demonstrate that health information–seeking behavior involves the action of seeking out information, irrespective of how or why it is sought [36].

Consistent with Mahmood et al [59], education and age are important sociodemographic factors associated with mHealth app use among people with obesity. As access to health services increases through the use of telehealth technology embedded within mHealth apps, it is imperative that this population group be able to benefit from this type of health service [60]. People with lower education levels and older individuals experience more limited eHealth literacy skills and lower mHealth app use, further widening the digital divide gap [18,24]. Additionally, when we examine other sociodemographic factors, marginalized ethnic groups such as Hispanic populations may have access to mHealth apps but experience digital divide issues [30,61].

Issues such as use and knowledge as they relate to using mHealth apps can also contribute to the digital divide [62]. Additional attention is needed to focus on these vulnerable populations. Interventions that can attempt to address this issue are the development of apps and health promotion campaigns that are designed to be culturally relevant [63]. Within the realm of health promotion and wellness, mHealth mindfulness approaches have been used for African American populations [64]. Moreover, studies have described the importance of mHealth interventions with phone features that are familiar to the target population group [62]. There should be consideration of acceptability and efficacy during the developmental phases to support the use of mHealth apps. When considering efficacy, simpler solutions in app design and use should be evaluated. For the older population, features such as 1-click access to a dashboard within health apps that are appropriately displayed in size may be appropriate. Future mHealth apps should also consider health education–related features to support users with low eHealth literacy skills [30,65]. The recent COVID-19 pandemic highlighted the continued digital divide and the disparity in health care services for those who lack sufficient digital literacy skills [66]. mHealth apps benefit people with various chronic conditions, including obesity. People with obesity are less likely to benefit from these mHealth apps if they have low eHealth literacy skills.

mHealth apps facilitate access to health information that has increasingly migrated to web-based spaces [67]. More importantly, mHealth devices assist individuals with seeking health information and decision-making regarding their health [68]. mHealth apps are also advantageous to improve access to health information for personal health data management [15,69]. Since we found that the eHealth literacy skills dimension of access for people with obesity is associated with higher odds of using mHealth apps for seeking health information for themselves, health services should reconsider how they disseminate health information to reach higher proportions of the population. Inevitably, the accessibility of web-based health information has changed the way people engage in health decision-making [70]. This is also evident from our network analysis results, which demonstrate the interconnectivity among all elements relating to eHealth literacy skills and mHealth app use among people with obesity, resulting in the need for holistic solutions to enhance mHealth app use and access to health information. Lenz’s model primarily focuses on the search process and use of the information; however, future studies should consider the nuanced contextual factors for people with obesity and their use of mHealth-related devices.

Accessing health information through mHealth apps streamlines the application of health information for decision-making. Many people with obesity have additional chronic diseases that can benefit from timely communication with their health care provider [71]. Effective communication is important for reported satisfaction and perceived health management outcomes. Face-to-face communication has been the standard for communication among patients and health care providers. However, there are mixed results on the perceived effectiveness of face-to-face communication versus IT-aided communication such as mHealth devices [72]. Recent studies have found that mHealth apps are viewed as useful by patients for improving communication and the accessibility of health data [73]. Therefore, this constant communication creates the potential for a bidirectional channel of communication among people with obesity and their health care providers. An in-depth content analysis of vaccination apps showed that few apps provide the capability for bidirectional communication among users and health care providers [74]. The challenges of bidirectional communication can be attributed to barriers to data integration. Given the numerous mHealth apps available for download, this creates interoperability challenges for electronic health care record systems [75]. For mHealth apps that are designed to improve physician and consumer communication, transdisciplinary scholarship is necessary to overcome these barriers. More importantly, technical and networking policies must be developed to support and incentivize the ability to improve this type of communication.

This study benefits from the use of a nationally representative sample of noninstitutionalized US adults. This study provides an adjusted analysis of the associations between mHealth app use and eHealth literacy skills among people with obesity. New technologies that require eHealth literacy skills are transforming how we receive health care and access health information, but they also highlight new disparities as they relate to digital health services [30]. However, to address the rise of chronic conditions such as obesity, it is essential to empower patients to engage in their own health management. One promising strategy is using mHealth apps as a complementary tool to manage weight loss and track physical activity [26]. We provide evidence of several significant factors that can be informative when designing inclusive mHealth app-based health intervention studies. Our results also have implications for studies aimed at managing weight loss or tracking the physical activity of people with obesity to assist with mHealth app development and uptake.

Concerning limitations, first, there could be additional confounding variables that are not included in the study, which is limited by the survey design questionnaire. Some of these confounding variables may be related to self-care behaviors or use patterns with mHealth apps [25]. Furthermore, a bias in the survey design includes the assumption that apps are used only on tablets or smartphones, such that only individuals who indicated having a tablet or smartphone were asked within the survey about having or using health or wellness apps. Second, respondents were only asked about access to information within the previous 12 months. There is a possibility that users do not access or seek health information between visits with their doctors on a yearly basis. Nevertheless, many patients with low health literacy are often left dissatisfied and unsure of the information shared by their doctor and seek third-party sources such as web-based health communities to fill those gaps [76,77]. Also, respondents who report ownership of a tablet, smartphone, or both may also use a computer, but they did not indicate that as part of their response. Third, results from the Ising model visualization show a strong relationship between respondents seeking health information for themselves and mHealth app use. As a result of seeking health information, this may also explain the strong relationship between mHealth app use and talking with one’s doctor. Therefore, these correlations may exist because they are measuring the same events. Also, some sociodemographic variables used in this study are correlated (eg, age and retired status), so some multicollinearity may be present. Fourth, with a small sample of uninsured people represented in our sample, the statistical significance for health insurance in our model may have been different with a larger sample of uninsured people. Additionally, the data did not provide a distinction between private and public health insurance, though the information content of such a factor may already be embedded in the income variable. A study aimed at self-monitoring of diet, physical activity, and weight among patients who were underinsured or uninsured demonstrated higher adherence through the use of 2 mHealth-related apps in comparison to a paper group [6]. Also, the sample in this study covers the COVID-19 peak period in early 2020, which may have represented a crest (and potentially a permanent shift in behaviors) in electronic access to health information among people with obesity compared to previous time periods. Lastly, there is a limitation in the HINTS survey questions as they were not designed using a web-based health information–seeking behavior framework, though we were able to detect relevant associations even with this design limitation.

Future directions of this work should consider this model structure for people without obesity. A comparative analysis may identify whether eHealth literacy relevance differs between individuals with obesity and those without obesity. This work also considered mHealth app use, but it did not examine cognitive motivational factors for mHealth use. The identification of motivational barriers and facilitators can be analyzed within the context of psychological motivation frameworks to identify potential intervention targets to leverage in mHealth intervention–based studies. Also, since the COVID-19 pandemic may have brought behavioral changes in the overall population regarding eHealth literacy, a dynamic study that explores those changes over time could highlight whether segments of people with obesity may now experience heightened needs compared to prepandemic stages.

### Conclusion

This study estimated the associations between mHealth app use and eHealth literacy skills. Our findings are consistent with previous literature, showing that eHealth literacy skills are associated with accessing digital health information and the application of digital health services. For example, age is negatively associated with mHealth app use among people with obesity, with other sociodemographic factors also showing strong associations. This highlights substantial uneven access to eHealth information among people with obesity, potentially leading to disparities in health outcomes among sociodemographic groups. It is imperative that this phenomenon be further investigated as digital health–related services that involve the use of mHealth apps become more integrated into health care services and aim to reach wider segments of the population. A continued challenge is to engage people with chronic conditions such as obesity to use mHealth apps, especially older individuals with obesity and those with lower educational backgrounds. Our work provides evidence of factors associated with mHealth app use in relation to access and application. This work provides an initial understanding of mHealth app use and eHealth literacy skills among people with obesity, and future studies should identify equitable solutions for people with obesity (as well as other groups) and their use of mHealth apps.

## Appendix

Multimedia Appendix 1 Supplementary figures and table.

## Abbreviations

AUC: area under the curve
HINTS: Health Information National Trends Survey
IRB: institutional review board
mHealth: mobile health
OR: odds ratio
ROC: receiver operating characteristic
