# Supplementary material for: The Association of eHealth Literacy Skills and mHealth Application Use Among US Adults With Obesity: Analysis of Health Information National Trends Survey Data
Source: JMIR Mhealth Uhealth. 2024 Jan 10;12:e46656. doi: 10.2196/46656 (PMC10809169; doi:10.2196/46656)
Supplement: Multimedia Appendix 1 [file mhealth_v12i1e46656_app1.docx]

**Supplementary Materials**

**Table S1.** Percentage of missing observations corresponding to each of the covariates in the full dataset (N=1,005). Rows represent percentages of missing observations by (non-missing) category of each variable. For example, for employment status, 3.88% of those declaring to be employed had incomplete data across other variables, while 18.29% of those declaring to be disabled had incomplete data.

|  |  | Total | Missing | % Missing |
| --- | --- | --- | --- | --- |
| **Outcome** | |  |  |  |
| mHealth App Use | No | 564 | 78 | 13.82 |
|  | Yes | 441 | 0 | 0.00 |
| **Main Covariates** | |  |  |  |
| ***eHealth Literacy Skills: Access Dimension*** | |  |  |  |
| Electronic Health Information for Self | No | 245 | 35 | 14.29 |
|  | Yes | 757 | 40 | 5.28 |
| Electronic Test Result(s) | No | 510 | 53 | 10.39 |
|  | Yes | 489 | 19 | 3.89 |
| ***eHealth Literacy Skills: Application Dimension*** | |  |  |  |
| Electronic Communication with Doctor/Doctor’s Office | No | 458 | 47 | 10.26 |
|  | Yes | 542 | 26 | 4.80 |
| Made Appointment(s) Electronically | No | 478 | 46 | 9.62 |
|  | Yes | 523 | 28 | 5.35 |
| **Sociodemographic Factors (Other Covariates)** | |  |  |  |
| Age | | 996 | 69 | 6.93 |
| Health Insurance | Insured | 943 | 67 | 7.10 |
|  | Uninsured | 58 | 7 | 12.07 |
| Gender at Birth | Female | 600 | 47 | 7.83 |
|  | Male | 398 | 24 | 6.03 |
| Employment Status | Disabled | 82 | 15 | 18.29 |
|  | Employed | 515 | 20 | 3.88 |
|  | Homemaker | 27 | 1 | 3.70 |
|  | Multiple | 109 | 9 | 8.26 |
|  | Retired | 210 | 26 | 12.38 |
|  | Unemployed | 45 | 2 | 4.44 |
|  | Other | 12 | 0 | 0.00 |
| Marital Status | Married/Living as married or with romantic partner | 549 | 30 | 5.46 |
|  | Separated/Divorced | 194 | 14 | 7.22 |
|  | Single/Never married | 168 | 7 | 4.17 |
|  | Widowed | 77 | 10 | 12.99 |
| Education | ≤ 11 years | 61 | 12 | 19.67 |
|  | 12 years or completed high school | 194 | 19 | 9.79 |
|  | Post high school training other than college | 77 | 8 | 10.39 |
|  | Some college | 245 | 12 | 4.90 |
|  | College graduate | 248 | 6 | 2.42 |
|  | Postgraduate | 166 | 7 | 4.22 |
| Annual Household Income | <$10,000 | 57 | 6 | 10.53 |
|  | $10,000 to $14,999 | 52 | 3 | 5.77 |
|  | $15,000 to $19,999 | 45 | 8 | 17.78 |
|  | $20,000 to $34,999 | 120 | 7 | 5.83 |
|  | $35,000 to $49,999 | 134 | 1 | 0.75 |
|  | $50,000 to $74,999 | 183 | 7 | 3.83 |
|  | $75,000 to $99,999 | 131 | 2 | 1.53 |
|  | $100,000 to $199,999 | 202 | 4 | 1.98 |
|  | ≥$200,000 | 41 | 0 | 0.00 |
| Race/Ethnicity | Black or African American | 156 | 7 | 4.49 |
|  | Hispanic | 184 | 10 | 5.43 |
|  | Non-Hispanic Asian | 18 | 0 | 0.00 |
|  | Non-Hispanic Multiple Races | 34 | 0 | 0.00 |
|  | Non-Hispanic Pacific islander or American Indian | 9 | 0 | 0.00 |
|  | Non-Hispanic White | 564 | 21 | 3.72 |
| Census Region | Midwest | 164 | 9 | 5.49 |
|  | Northeast | 154 | 12 | 7.79 |
|  | South | 473 | 41 | 8.67 |
|  | West | 213 | 15 | 7.04 |

(**Figure S1.** Weighted boxplot of age by mHealth app use.)


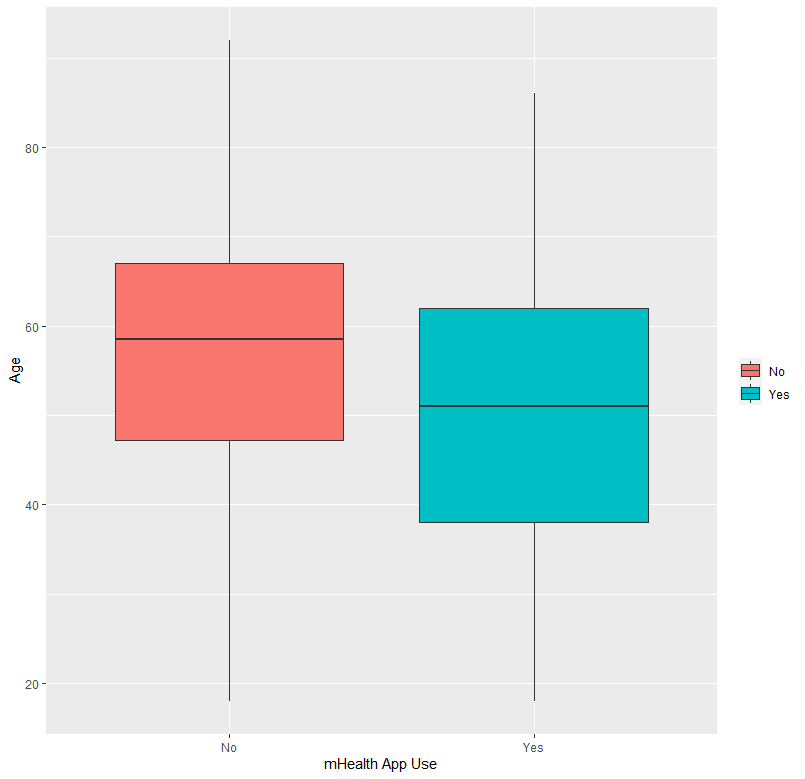


(**Figure S2.** 100% stacked bar chart for health insurance status and mHealth app use.)


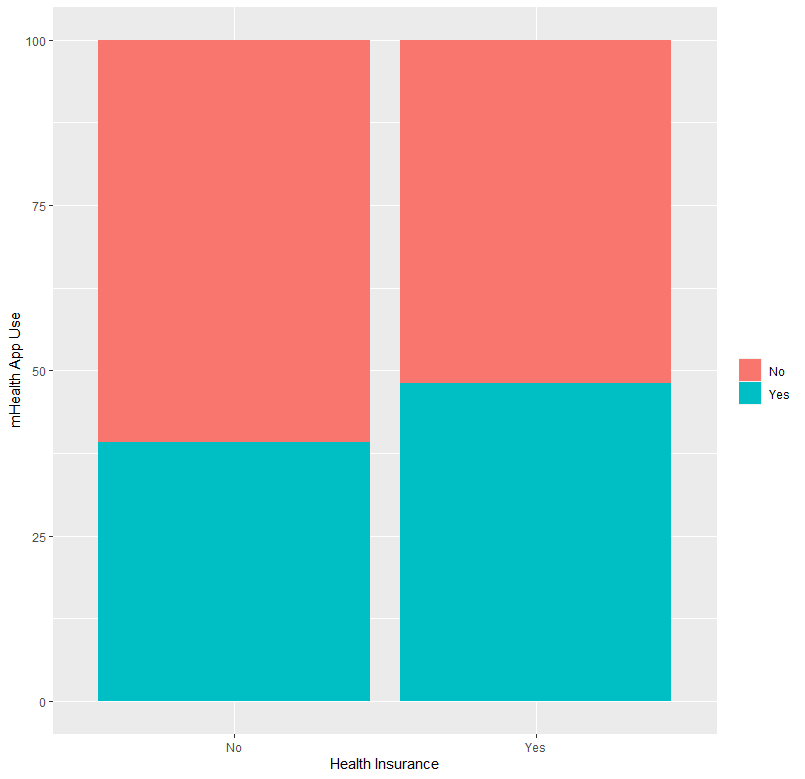


(**Figure S3.** 100% stacked bar chart for gender at birth and mHealth app use.)


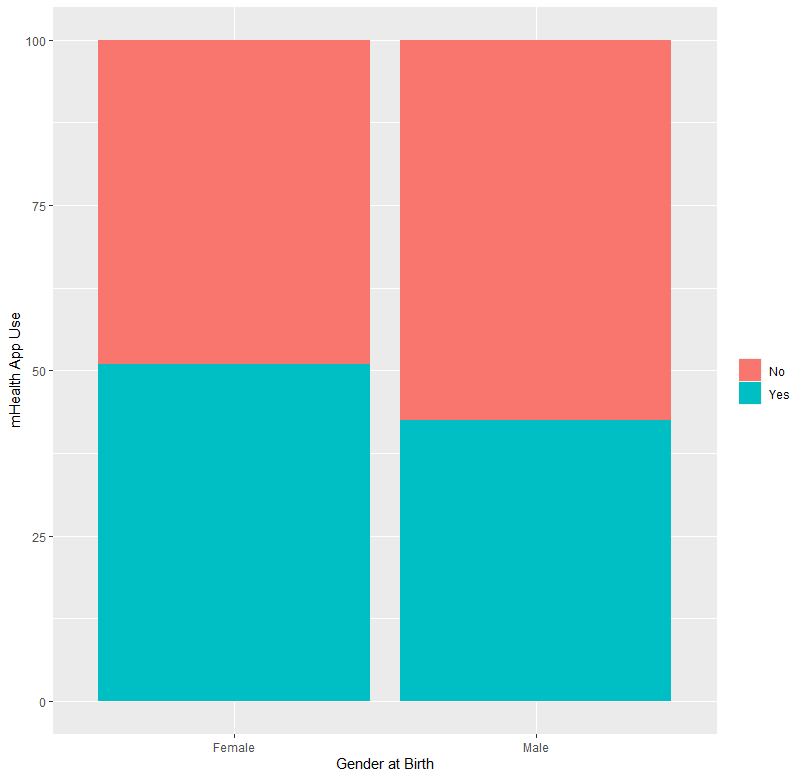


(**Figure S4.** 100% stacked bar chart for employment status and mHealth app use.)


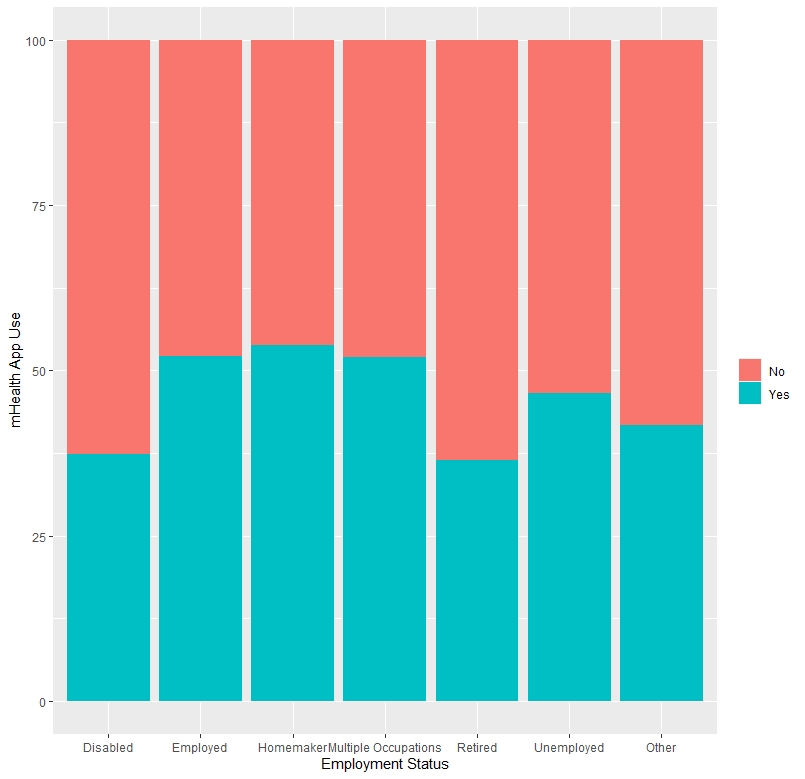


(**Figure S5.** 100% stacked bar chart for marital status and mHealth app use.)


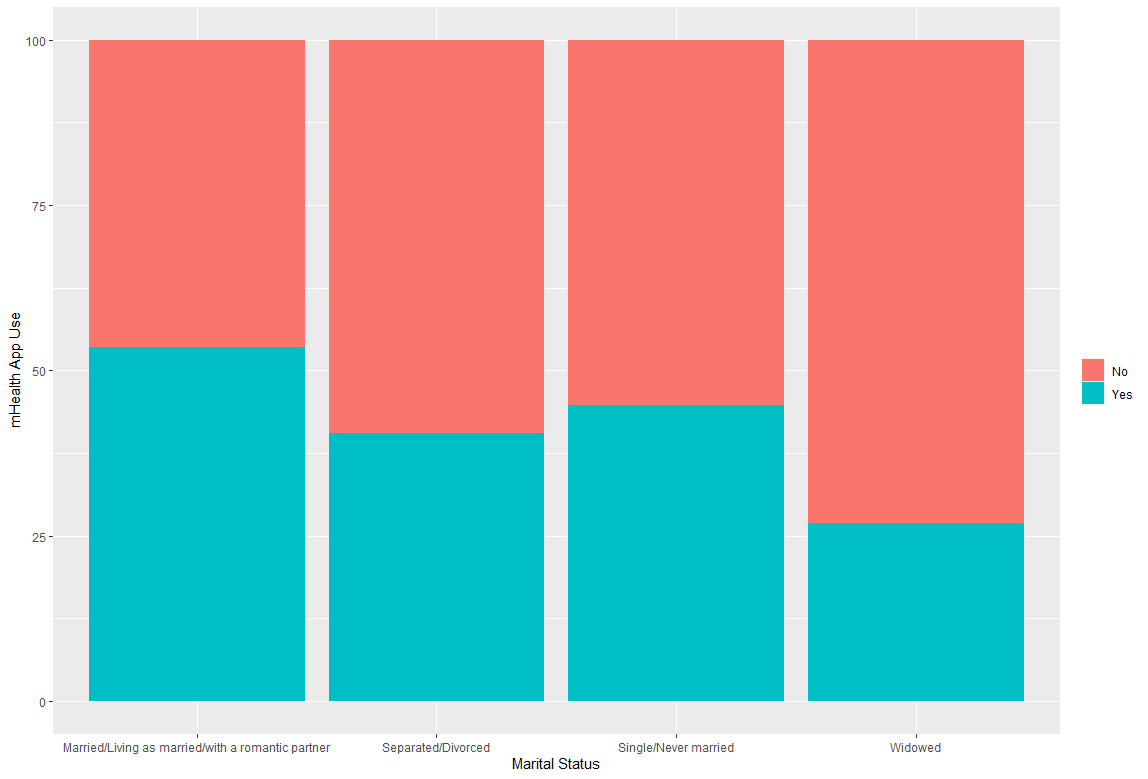


(**Figure S6.** 100% stacked bar chart for education and mHealth app use.)


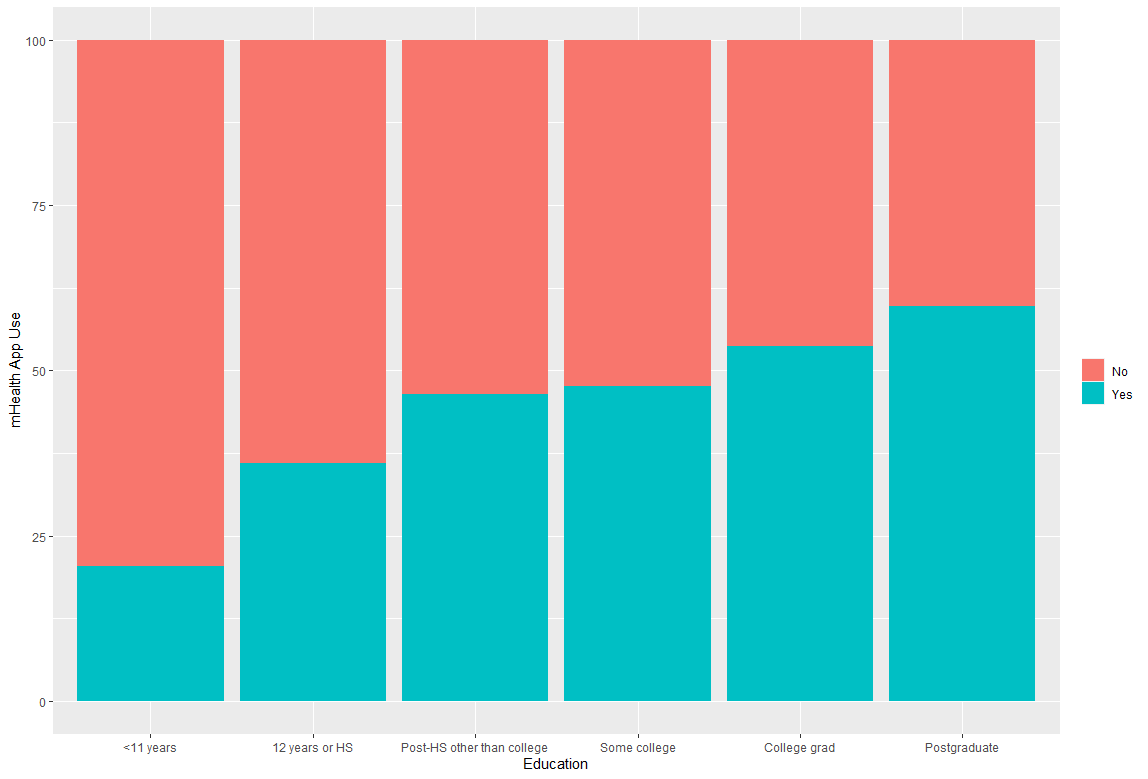


(**Figure S7.** 100% stacked bar chart for income and mHealth app use.)


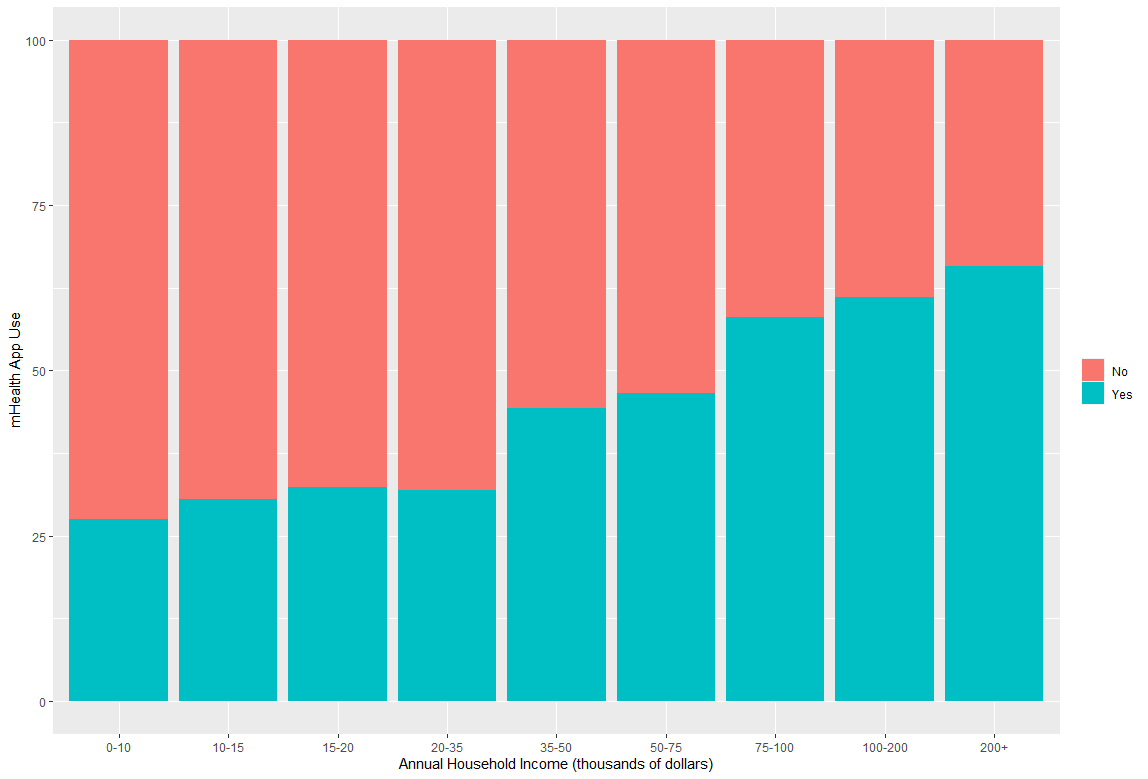


(**Figure S8.** 100% stacked bar chart for race/ethnicity and mHealth app use.)


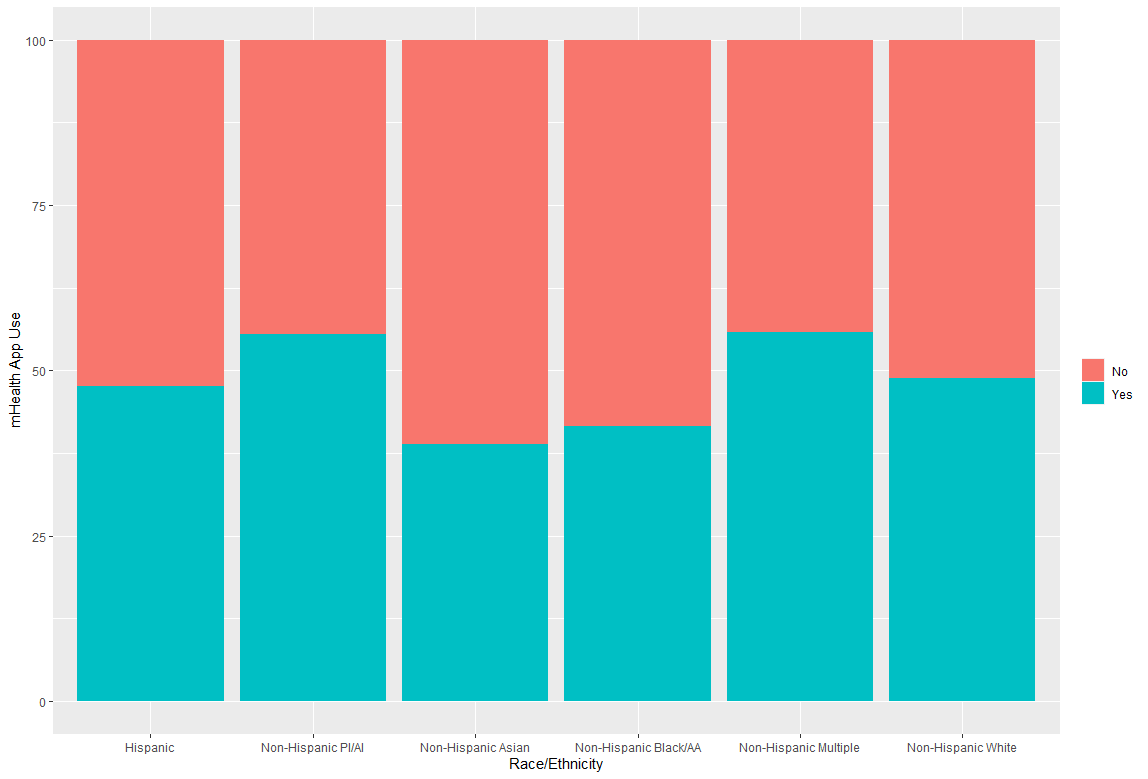


(**Figure S9.** 100% stacked bar chart for Census region and mHealth app use.)


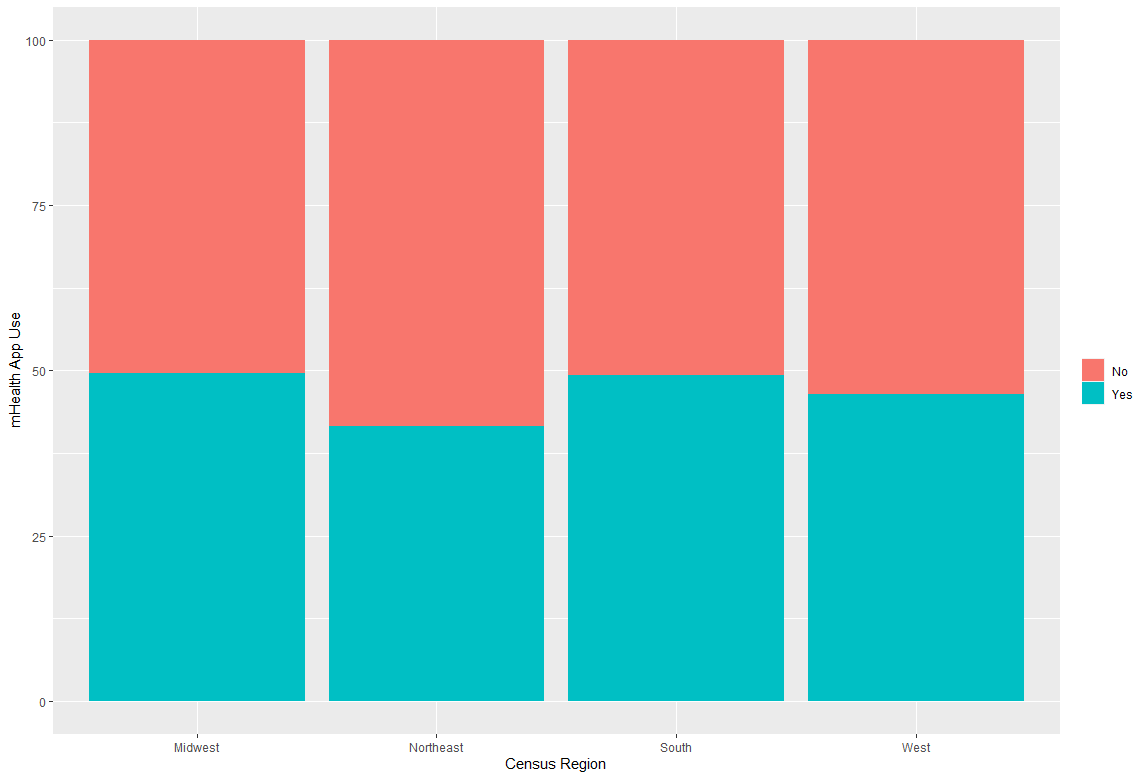


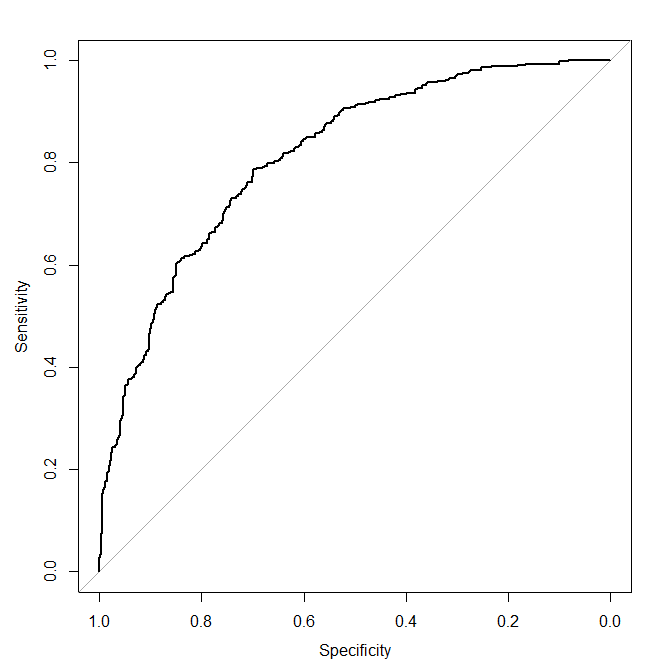
(**Figure S10.** Receiver operating curve (ROC) resulting from the multivariable logistic model using the primary complete case analysis.)
